# Supplementary material for: Towards unified AI-driven fracture mechanics: the extended deep energy method (XDEM)
Source: Nat Commun. 2026 Aug 15;17:8492. doi: 10.1038/s41467-026-76748-1 (PMC13484471; doi:10.1038/s41467-026-76748-1)
Supplement: Supplementary file 3 [file 41467_2026_76748_MOESM3_ESM.zip › pic/discussion/virtural_pattern/crack_virtual.pdf]

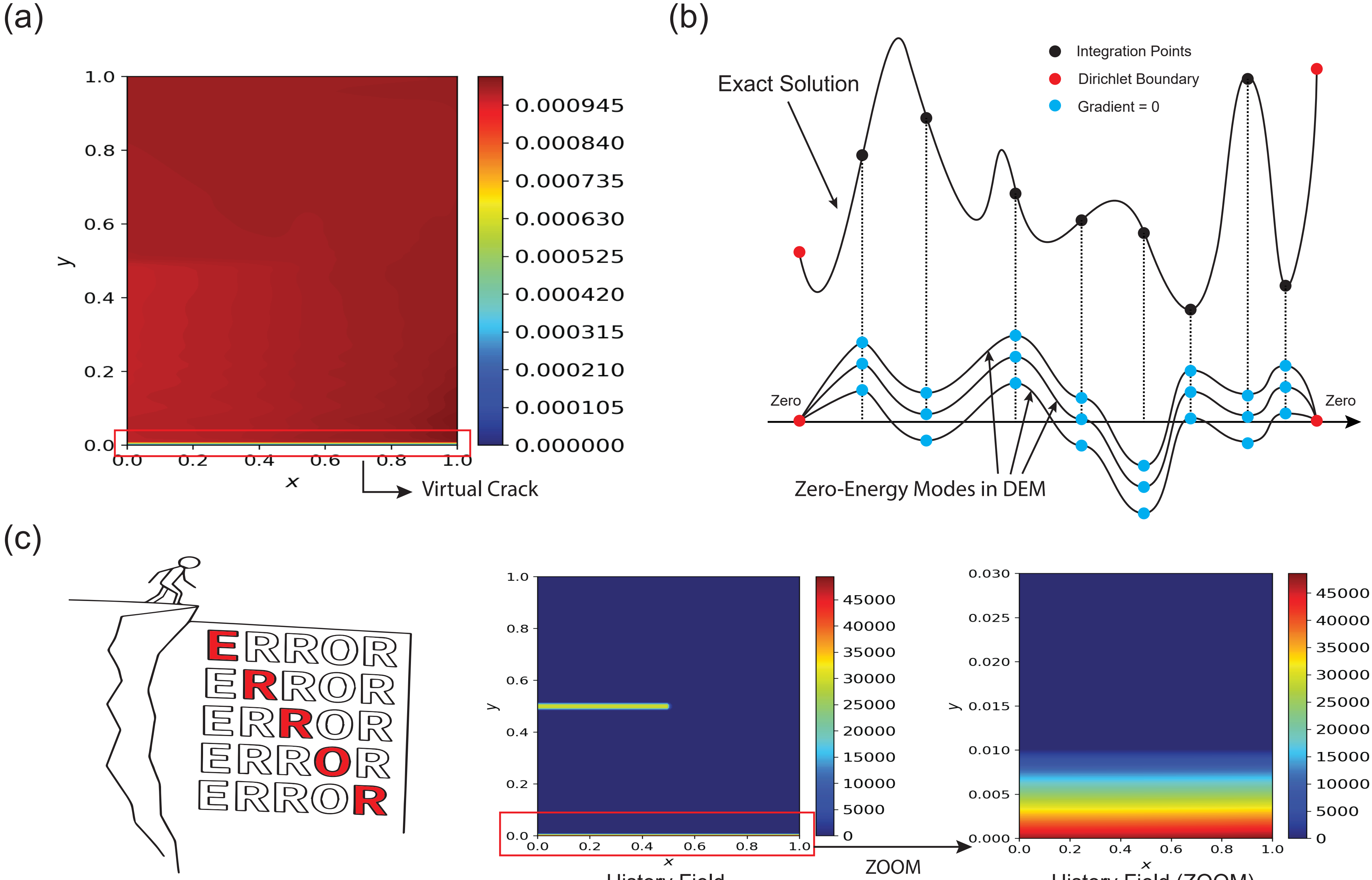

(a)
1.0
0.8
0.6
0.4
0.2
0.0
y
0.0 0.2 0.4 0.6 0.8 1.0
x
0.000945
0.000840
0.000735
0.000630
0.000525
0.000420
0.000315
0.000210
0.000105
0.000000
Virtual Crack
(b)
Exact Solution
Integration Points
Dirichlet Boundary
Gradient = 0
Zero
Zero
Zero-Energy Modes in DEM
(c)
ERROR
ERROR
ERROR
ERROR
ERROR
1.0
0.8
0.6
0.4
0.2
0.0
y
0.0 0.2 0.4 0.6 0.8 1.0
x
45000
40000
35000
30000
25000
20000
15000
10000
5000
0
History Field
ZOOM
0.030
0.025
0.020
0.015
0.010
0.005
0.000
y
0.0 0.2 0.4 0.6 0.8 1.0
x
45000
40000
35000
30000
25000
20000
15000
10000
5000
0
History Field (ZOOM)
